# The effect of flammer-syndrome on retinal venous pressure

**DOI:** 10.1186/1471-2415-14-121

**Published:** 2014-10-13

**Authors:** Lei Fang, Michael Baertschi, Maneli Mozaffarieh

**Affiliations:** Department of Ophthalmology, University of Basel, Mittlere Strasse 91, 4031 Basel, Switzerland; Department of Biomedicine, University of Basel, Basel, Switzerland

## Abstract

**Background:**

The purpose of the study was to measure the retinal venous pressure (RVP) in the eyes of primary open-angle glaucoma (POAG) patients and healthy subjects with and without a Flammer-Syndrome (FS).

**Methods:**

RVP was measured in the following four groups of patients and age- and sex-matched healthy controls: (a) 15 patients with a POAG and a FS (POAG/FS+); (b) 15 patients with a POAG but without a FS (POAG/FS-); (c) 14 healthy subjects with a FS (healthy/FS+) and (d) 16 healthy subjects without a FS (healthy/FS-). RVP was measured in all participants bilaterally by means of contact lens ophthalmodynamometry. Ophthalmodynamometry is done by applying increasing pressure on the eye via a contact lens. The minimum force required to induce a venous pulsation is called ophthalmodynamometric force (ODF). The RVP is defined and calculated as the sum of ODF and intraocular pressure (IOP) [RVP = ODF + IOP].

**Results:**

The participants with a FS (whether patients with POAG or healthy subjects), had a significantly higher RVP compared to subjects without a FS (p = 0.0103). Patients with a POAG and FS (POAG/FS+) had a significantly higher RVP compared to patients without a FS (POAG/FS-) (p = 0.0301). There was a notable trend for a higher RVP in the healthy/FS + group compared to the healthy/FS - group, which did not reach statistical significance (p = 0.0898).

**Conclusions:**

RVP is higher in subjects with a FS, particularly in glaucoma patients. The causal relationship needs to be further evaluated.

## Background

Disturbances of ocular blood flow are involved in many ophthalmic diseases and are therefore of utmost clinical relevance [1–5]. There are various causes for blood flow disturbances, such as diseased blood vessels [6] or mechanical compression of the vessel wall [7]. However, some organs are not well perfused, despite anatomically healthy blood vessels, when the regulation of blood flow is not adapted to the needs of the tissue [8]. Such a vascular dysregulation implies either inappropriate vasoconstrictions (vasospasms) or an insufficient vasodilation (more or less than is required) [9]. Dysregulation can be secondary in nature, as in multiple sclerosis [10], wherein the high level of Endothelin-1 reduces ocular blood flow OBF. Dysregulation can also be primary in nature (primary vascular dysregulation or PVD) [9], meaning that it can occur without any underlying disease and caused by an inborn tendency to respond differently to various stimuli, such as cold temperatures or mechanical or emotional stress. The combination of PVD with a cluster of additional vascular and non-vascular signs and symptoms is what is known today as the Flammer-Syndrome [11, 12].

The eye is one of the best-perfused organs in the body. One factor influencing this process is the ocular perfusion pressure (OPP) [13–17]. OPP is the difference between systemic blood pressure and the RVP. In the eye, arterial pressure is assumed to be 2/3 of the brachial arterial pressure. The RVP is assumed equal to the IOP. The latter assumption is not always true in glaucoma patients [18–21].

As summarized in the literature reviews, glaucoma patients often concomitantly suffer from a FS [22, 23]. One of the clinical observations that we made in patients with a FS was that they often had dilated retinal veins, which is why we hypothesized that RVP may be higher in FS than in non-FS subjects. We therefore set out to measure RVP in glaucoma patients and healthy subjects with and without a FS.

## Methods

Patients with POAG were recruited from the University Eye Clinic, Basel, between January 2011 and December 2012. Healthy volunteers, age- and sex-matched to the POAG patients, were recruited in our outpatient department. The control subjects did not have any relevant eye disease and attended our outpatient department for various reasons, including prescriptions for eyeglasses, dry eye symptoms and regular ophthalmic check-up examinations. Ethical approval was obtained from the local medical ethics committee of Basel City (‘Ethik Kommission Beider Basel’ or EKBB) to measure RVP in healthy controls who gave oral consent to take part in the study (Reference Number 272/11). No ethical approval was required to measure RVP in glaucoma patients as RVP measurements are always taken in all glaucoma patients at the Department of Ophthalmology of the University of Basel. For inclusion, the patients with POAG met the following criteria: (1) glaucomatous visual fields or glaucomatous optic nerve cupping and (2) the absence of alternative causes of optic neuropathy.

FS was defined as being present if it was detected in the patient history and confirmed by the dynamic retinal vessel analyser (DVA). Cases in which the patient history and DVA results were contradictory were excluded from the study.

### Evaluation of patient history for FS

FS is defined as present (FS+) in the patient history if the subjects answer three of the following six questions with “Yes”, and it is defined as absent (FS-) if the subjects answer less than three questions with “Yes”: 1) Do you suffer from cold hands or feet even in summer [24]?; 2) Do you have trouble falling asleep, especially when you are cold [25]?; 3) Are you seldom thirsty, and do you have to remind yourself to drink enough [26]?; 4) Do you suffer from migraine attacks [27]?; 5) Do you have low blood pressure [28]?; 6) Do you identify smells better than others [29]?

### Evaluation of DVA results for FS

The results of DVA were considered positive for FS (pathological) if the reaction of the arteries in both eyes was reduced in response to flickering light.

Cases in which the patient history and DVA results were contradictory were excluded from the study. The following groups of subjects were compared: (1) POAG patients with a FS (POAG/FS+); (2) POAG patients without a FS (POAG/FS-); (3) healthy controls with a FS (healthy/FS+) and (4) healthy controls without a FS (healthy/FS-). Table 1 presents the demographic data of the different groups of subjects. Table 2 lists the local and systemic treatment regimens of the POAG patients.

**Table 1:** **Demographic and baseline characteristics of the four groups of participants**

	POAG/FS+	POAG/FS-	Healthy/FS+	Healthy/FS-
N	15	15	14	16
Gender (F/M)	8/7	7/8	7/7	10/6
Age Mean (SD)	67.0 (8.7)	62.8 (8.7)	60.4 (13.2)	56.5 (10.6)
IOP Mean (SD)	10.6 (1.5)	13.33 (2.55)	11.71 (1.33)	13.12 (3.3)

Patients with POAG and FS: POAG/FS +.

Patients with POAG but without FS: POAG/FS -.

Healthy subjects with FS: Healthy/FS + .

Healthy subjects without FS: Healthy/FS -.

**Table 2:** **List of local and systemic treatment regimens of the POAG patients**

Local & systemic therapy	POAG/FS+	POAG/FS-
**Local Therapy N**	15	15
Timolol & Dorzolamide	1	0
Timolol & Dorzolamide & Tafluprost	4	5
Betaxolol & Dorzolamide, Tafluprost	2	3
Brinzolamide	1	0
Tafluprost	3	1
Latanoprost	0	3
No local Therapy	4	3
**Systemic Therapy N**	15	15
Ginkgo biloba (120 mg daily)	1	2
Ginkgo biloba (120 mg daily) & Magnesium (10 mmol daily)	6	3

For all patients and controls, RVP was measured in both eyes by ophthalmodynamometry (Meditron GmbH, Völklingen, Germany). This device consists of a conventional Goldmann contact lens fitted with a pressure sensor at its outer margin where the Goldmann contact lens is usually held during an ophthalmoscopic examination. The device is connected to an LCD screen.

Ophthalmodynamometry is conducted by applying increasing pressure to the eye via the contact lens. This applied pressure can be read as an IOP increase on the attached LCD screen based on a calibration curve. The IOP increase that is required to induce a venous pulsation is called the ophthalmodynamometric force (ODF). If a spontaneous venous pulsation is present, ODF is said to be 0, if not present, increasing pressure is applied. The RVP is defined and calculated as the sum of the ODF and IOP [RVP = ODF + IOP]. Measurements by the ophthalmodynamometer are reproducible [30].

### Statistical analysis

RVP was analysed with a linear fixed effects model. The participant group (POAG/FS+, POAG/FS-, healthy/FS + and healthy/FS-) was taken as ‘fixed effect’, and participants (patients and healthy controls) were taken as ‘random effects’ to account for repeated measures. Gender and age were included as covariates to account for baseline differences. The RVP was log-transformed to meet the assumption of normally distributed errors. Three a priori defined group comparisons were made: FS + vs FS-, POAG/FS + vs POAG/FS - and Healthy/FS + vs Healthy/FS -.

## Results

The visual acuities in the FS + group ranged from 20/100 to 20/20 and the mean deviation (MD) in the visual field ranged from -3.2 to -10.9. In the FS- group the visual acuities ranged from 20/100 to 20/20 and MD in the visual field ranged from -2.9 to -12.1.

Five of the patients in the POAG/FS + group and two patients in the POAG/FS- group had low blood pressure, but neither of these patients was treated with salt tablets or fludrocortisone at the time when RVP was measured.

**Figure 1:**
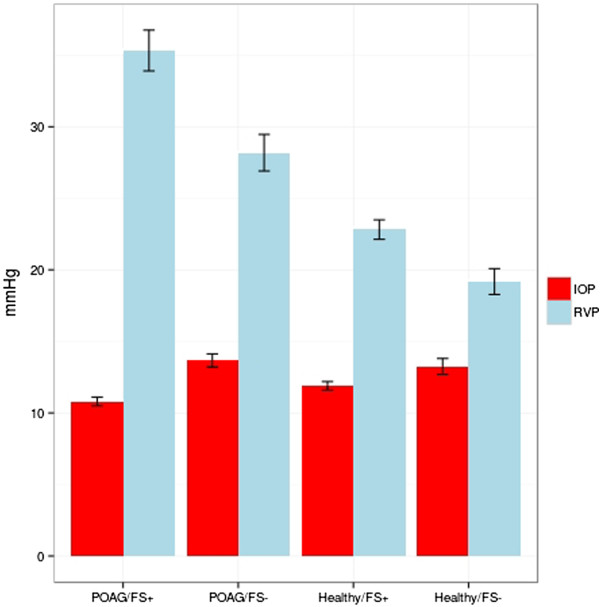
**Intraocular pressure (IOP) and retinal venous pressure (RVP) in the four groups of participants: for IOP mean ±1 standard deviation of base line measurements are shown; for RVP mean ±1 standard error are shown.**

Participants with a FS (whether patients with a POAG or healthy subjects), had a significantly higher RVP compared to participants without a FS (P = 0.0103). Patients with a POAG and FS had a statistically significant higher RVP compared to patients with a POAG but without FS (p = 0.0301, Figure 1).

There was a notable trend for a higher RVP in the healthy/FS + group compared to the healthy/FS - group, which did not reach statistical significance (p = 0.0898). RVP of healthy subjects was on average 23% higher compared to healthy subjects without FS (Figure 1).

No significant differences in RVP were found between the two eyes of all participants (Table 2).

## Discussion

The term vascular dysregulation in the context of glaucoma was first introduced by Flammer [9]. Later, a distinction was made between primary and secondary vascular dysregulation [31]. A secondary vascular dysregulation occurs in the context of another disease, such as rheumatoid arthritis [32] whereas a primary vascular dysregulation [2], described an inborn disposition to respond differently to stimuli. Today, the combination of PVD with a cluster of additional vascular and non-vascular signs and symptoms is called the Flammer-Syndrome (FS) [11, 12]. The FS can occur in otherwise healthy subjects. Since we made the clinical observation that glaucoma patients with a FS often had dilated retinal veins, we hypothesized that the RVP in the subset of glaucoma patients with a FS may be higher than in those glaucoma patients without a FS.

Glaucoma patients have a higher RVP than controls [18–21, 33]. Our values of RVP in the glaucoma group without FS (POAG/FS-) were in line with those of Jonas et al. who measured the diastolic collapse pressure of the central retinal vein in their glaucoma group to be 26.1 (SD 26.4) relative units [19]. Our results suggest that glaucoma patients who suffer from a FS have a significantly higher RVP than non-FS glaucoma patients. The vascular systems of people with a FS respond differently (e.g., reacting with vasoconstrictions to various stimuli such as cold or stress) [34, 35]. Despite the anatomically normal appearance of their vessels, those people with a FS have stiffer retinal vessels, as pulse waves in their retinal vessels propagate faster compared to those of subjects without a FS [36]. The spatial irregularity of the vessels of people with a FS is increased [37], whereas neurovascular coupling is decreased [38], and autoregulation of ocular blood flow is disturbed [35].

A reduced and unstable OPP has been reported to be risk factor for glaucoma progression [13, 15, 39–41]; therefore, a better estimate of OPP obtained by considering RVP may reveal an even stronger relationship. At present, the cause of this increased RVP is not known. Theoretically, it could be due to structural changes in the optic nerve head or to a local dysregulation at the outflow level of the retinal vein, as already postulated for the mechanism of a retinal vein occlusion [42]. Such dysregulation is most likely a consequence of the local increase of vasoactive molecules, such as Endothelin-1, which are diffused from the circulating blood or are produced in the neural tissue of the retina [43]. Endothelin-1 values are higher in glaucoma patients, particularly normal-tension glaucoma patients who commonly suffer from a FS, compared to healthy controls [44–46].

This study has certain limitations. Central corneal thickness was not measured as it is still a debate as to whether it is meaningful to correct IOP by corneal thickness. Caffeine consumption was also not assessed in this study since the consumption of caffeine does not seem to impact values of IOP or ocular perfusion pressure (OPP) for those at risk for POAG [47]. The patients in the FS + and FS- groups were also not fully matched with regards to the extent of glaucomatous damage, length or disease or local and systemic treatment.

## Conclusion

In summary, FS appears to be associated with an increased RVP particularly in glaucoma patients. The causal relationship needs to be further evaluated.
